# Multi-laboratory validation study of multilocus variable-number tandem repeat analysis (MLVA) for *Salmonella enterica* serovar Enteritidis, 2015

**DOI:** 10.2807/1560-7917.ES.2017.22.9.30477

**Published:** 2017-03-02

**Authors:** Tansy Peters, Sophie Bertrand, Jonas T Björkman, Lin T Brandal, Derek J Brown, Tímea Erdõsi, Max Heck, Salha Ibrahem, Karin Johansson, Christian Kornschober, Saara M Kotila, Simon Le Hello, Taru Lienemann, Wesley Mattheus, Eva Møller Nielsen, Catherine Ragimbeau, Jillian Rumore, Ashley Sabol, Mia Torpdahl, Eija Trees, Alma Tuohy, Elizabeth de Pinna

**Affiliations:** 1 Public Health England, London, United Kingdom; 2 Scientific Institute of Public Health, Brussels, Belgium; 3 Statens Serum Institut, Copenhagen, Denmark; 4 Norwegian Institute of Public Health, Oslo, Norway; 5 Scottish Microbiology Reference Laboratories, Glasgow, United Kingdom; 6 National Center for Epidemiology, Budapest, Hungary; 7 National Institute for Public Health and the Environment, Bilthoven, The Netherlands; 8 National Institute for Health and Welfare, Helsinki, Finland; 9 European Centre for Disease Prevention and Control (ECDC), Solna, Sweden; 10 Austrian Agency for Health and Food Safety, Graz, Austria; 11 Institut Pasteur, Paris, France; 12 Laboratoire National de Santé, Dudelange, Luxembourg; 13 Public Health Agency of Canada, Winnipeg, Canada; 14 Centers for Disease Control and Prevention, Atlanta, USA; 15 University Hospital Galway, Galway, Ireland

**Keywords:** food-borne infections, Salmonella, typing, laboratory, laboratory surveillance

## Abstract

Multilocus variable-number tandem repeat analysis (MLVA) is a rapid and reproducible typing method that is an important tool for investigation, as well as detection, of national and multinational outbreaks of a range of food-borne pathogens. S*almonella enterica* serovar Enteritidis is the most common *Salmonella* serovar associated with human salmonellosis in the European Union/European Economic Area and North America. Fourteen laboratories from 13 countries in Europe and North America participated in a validation study for MLVA of *S.* Enteritidis targeting five loci. Following normalisation of fragment sizes using a set of reference strains, a blinded set of 24 strains with known allele sizes was analysed by each participant. The *S.* Enteritidis 5-loci MLVA protocol was shown to produce internationally comparable results as more than 90% of the participants reported less than 5% discrepant MLVA profiles. All 14 participating laboratories performed well, even those where experience with this typing method was limited. The raw fragment length data were consistent throughout, and the inter-laboratory validation helped to standardise the conversion of raw data to repeat numbers with at least two countries updating their internal procedures. However, differences in assigned MLVA profiles remain between well-established protocols and should be taken into account when exchanging data.

## Introduction

The global public health impact of non-typhoidal salmonellosis is high, with an estimated 93.8 million illnesses, of which 80.3 million are estimated to be food-borne [1].

The ability to rapidly identify the primary sources of bacterial contamination using genetic subtyping is critical in the investigation of food-borne infections. If common outbreak sources can be determined in a timely fashion, further *Salmonella* infections can be prevented.

Multilocus variable-number tandem repeat (VNTR) analysis (MLVA) is a rapid, inexpensive and reproducible high-resolution typing method that has become an increasingly popular tool for the investigation, as well as detection, of national and multinational outbreaks of a range of foodborne pathogens [2-6].The method is based on multiplex PCR amplification of repetitive DNA elements organised in tandem within the genome (tandem repeats), followed by concurrent fragment size analysis of the resulting amplified polymorphic regions. The latter are detected using capillary electrophoresis (CE) where an internal size standard is included for each sample. *Salmonella enterica* serovar Typhimurium (*S.* Typhimurium) MLVA, using five loci known to demonstrate inter-strain variability, has previously been validated successfully during inter-laboratory comparisons [7,8]. The resulting protocol [9] is used by countries in the European Union (EU) and European Economic Area (EEA) that report molecular data to The European Surveillance System (TESSy) [10].

However, *Salmonella enterica* serovar Enteritidis (*S.* Enteritidis) remains the most commonly reported serovar within the EU/EEA. In 2013, it was responsible for 39.5% of *Salmonella* infections in humans, followed by *S.* Typhimurium (20.2%) [11]. Due to the lack of genetic variation within the serovar Enteritidis population, previous molecular methods, such as pulsed-field gel electrophoresis (PFGE), lack the necessary discrimination for informing outbreak investigations. Thus the utility of MLVA has come to the fore.

A nine loci MLVA scheme for this serovar was originally developed by Hopkins et al. in 2011 [12], and has found widespread popularity within the EU. The Hopkins publication concluded that selecting fewer loci could also provide adequate discrimination, and exclusion of loci that showed minimal diversity left five specific loci remaining, all with relatively short repeats at 6–12 base pairs (bp). The Hopkins’ scheme nomenclature follows the same basic tenet as for *S.* Typhimurium MLVA [13] i.e. it is based on the actual number of repeats in each locus and the MLVA profile is described as a string of five numbers.

The publication of the Hopkins protocol triggered the independent development of many different protocols for *S.* Enteritidis MLVA by individual laboratories. The production of comparable data between laboratories is crucial for the usefulness of typing for foodborne pathogens, thus, there was a need to harmonise the current MLVA methodologies for *S.* Enteritidis and reach consensus with regard to nomenclature, comparability and meaningful interpretation of data.

Using recommendations provided by previous MLVA harmonisation studies [7,14], the objective of the present study was to test whether comparable *S*. Enteritidis MLVA results could also be obtained between different laboratories, often using different equipment. Study participants were provided with a suggested MLVA protocol but were not obliged to use this and could follow any in-house protocols that already existed within their laboratory. However, they were all asked to analyse the same five loci, in the same order and report the number of tandem repeats found at each locus.

## Methods

This international, inter-laboratory comparison of MLVA results was largely based upon the recommendations of Nadon et al. [14] for intra- and inter-laboratory validation of MLVA schemes and was carried out using a set of calibration strains to redress any laboratory or equipment set-up-dependent discrepancies between sequenced and measured fragment lengths. Following the initial set-up and normalisation of fragment sizes for the calibration set, 14 laboratories from 13 different countries participated in an inter-laboratory validation of MLVA for *S.* Enteritidis using a blinded set of 24 strains with known allele sizes.

### Participants

Fourteen laboratories (A–N), 12 from EU/EEA countries and two from North America (Canada and the United States (US)), participated in the validation, most using a scheme routinely used in their own laboratory for *S.* Enteritidis MLVA. Although largely a European initiative, it was important to ensure global comparability of typing results and therefore participants from Canada and the US were invited to take part in this study. The participants comprised 13 national public health laboratories and one national public health and food safety laboratory. Participants’ experience in *S.* Enteritidis MLVA varied from having only recently set up the method to having performed extensive validations of the method over the years.

### Bacterial isolates

Using differing CE platforms and chemistries is known to yield different fragment sizes which in turn may affect the interpretation of the correct number of tandem repeats as determined by sequencing. To overcome this, each laboratory was firstly required to calibrate their own equipment using a set of 16 reference strains with sequenced alleles [12]. Strains were selected from Public Health England’s (PHE) collection of isolates to provide a good coverage of the range of alleles known to exist at each locus. The five loci chosen were SENTR4, SENTR5, SENTR6, SENTR7 and SE-3 [15]; alternate names [16,17], bp lengths and number of tandem repeats are shown in Table 1. These *S*. Enteritidis strains enabled laboratories to normalise their raw fragment data to actual fragment sizes.

**Table 1:** Reference strains for MLVA of *Salmonella enterica* serovar Enteritidis (adapted from Hopkins et al. [12])

Calibration strain	SENTR7 (SE9, STTR9)		SENTR5 (SE5, STTR5)		SENTR6 (SE2, ENTR20)		SENTR4 (SE1, ENTR13)		SE-3	
	Length in bp^a^	Number of TRs	Length in bp^a^	Number of TRs	Length in bp^a^	Number of TRs	Length in bp^a^	Number of TRs	Length in bp^a^	Number of TRs
HPA001	135	3	265	9	173	4	119	4	308	1
HPA002	135	3	301	15	180	5	119	4	320	2
HPA003	126	2	277	11	180	5	112	3	320	2
HPA004	135	3	289	13	180	5	119	4	309	1
HPA005	135	3	271	10	187	6	119	4	309	1
HPA006	117	1	265	9	194	7	112	3	320	2
HPA007	126	2	295	14	208	9	112	3	320	2
HPA008	126	2	277	11	215	10	112	3	320	2
HPA009	126	2	233	12	229	12	112	3	320	2
HPA010	126	2	235	4	208	9	126	5	0	NA
HPA011	126	2	247	6	187	6	126	5	308	1
HPA012	126	2	253	7	229	12	133	6	308	1
HPA013	126	2	259	8	201	8	126	5	320	2
HPA014	126	2	271	10	236	13	126	5	308	1
HPA015	126	2	301	15	201	8	140	7	0	NA
HPA016	126	2	253	7^b^	194	7	147	8	0	NA

bp: base pairs; MLVA: multilocus variable-number tandem repeat analysis; NA: no amplification at this locus; TR: tandem repeat.

^a^ Length of fragment as determined by sequencing, which may differ from the size determined by capillary electrophoresis.

^b^ Sequence of first three TRs is GACCAC-GACCAC-GGCCAT.

A further set of 21 isolates were chosen as a blinded validation set from ca 2,000 *S.* Enteritidis previously MLVA-typed at PHE (Table 2). The MLVA profiles for these are stored within a BioNumerics database at PHE and the validation set was selected to represent a wide range of the known allelic diversity at each of the five loci. Three of the isolates were included in duplicate to test the reproducibility and repeatability of the method making a total of 24 blinded isolates (ECDC1-ECDC24).

**Table 2:** Validation strain panel for the five-locus *Salmonella enterica* serovar Enteritidis multilocus variable-number tandem repeat analysis

Validation strain^a^	MLVA		
	Fragment sizes^b^	Profile (TRs)	Number of laboratories identifying incorrectly (incorrectly identified locus)
ecdc_1	131–297–176–118–317	3–15–5-4–2	0
ecdc_2	122–273–176–111–318	2–11–5-3–2	0
ecdc_3	131–285–176–118–305	3–13–5-4–1	Strain excluded
ecdc_4	131–267–183–118–305	3–10–6-4–1	1 (SENTR4)
ecdc_5	113–261–190–111–317	1–9-7–3-2	0
ecdc_6	122–291–204–111–317	2–14–9-3–2	0
ecdc_7	122–273–211–111–317	2–11–10–3-2	0
ecdc_8	122–231–204–125–0	2–4-9–5-NA	2 (SENTR5, SE-3)
ecdc_9	122–243–183–125–305	2–6-6–5-1	0
ecdc_10	122–249–226–133–305	2–7-12–6-1	1 (SENTR5)
ecdc_11	121–291–190–111–318	2–14–7-3–2	0
ecdc_12	121–260–196–117–317	2–9-8–4-2	0
ecdc_13	121–267–183–111–318	2–10–6-3–2	0
ecdc_14	131–255–176–118–305	3–8-5–4-1	2 (SENTR7, SENTR5)
ecdc_15	130–279–169–118–305	3–12–4-4–1	0
ecdc_16	112–273–190–111–317	1–11–7-3–2	0
ecdc_17	121–267–197–124–317	2–10–8-5–2	0
ecdc_18	121–297–203–110–317	2–15–9-3–2	0
ecdc_19	130–237–176–111–305	3–5-5–3-1	0
ecdc_20	122–279–161–111–317	2–12–3-3–2	0
ecdc_21	131–273–175–118–305	3–11–5-4–1	Strain excluded
ecdc_22	112–273–190–111–317	1–11–7-3–2	0
ecdc_23	121–267–197–124–317	2–10–8-5–2	1 (SENTR4)
ecdc_24	130–237–176–111–305	3–5-5–3-1	0

MLVA: multilocus variable-number tandem repeat analysis; NA: no amplification at this locus; TR: tandem repeat.

^a^ MLVA target alleles were sequenced for validation strains 1–10 and 20.

^b^ Length of fragment as determined by capillary electrophoresis at Public Health England using ABI 3730 DNA Analyzer (Applied Biosystems, Foster City, California, US), order of alleles SENTR7; SENTR5; SENTR6; SENTR4; SE-3.

Three samples were duplicated and therefore have identical profiles: ecdc_16 and ecdc_22; ecdc_17 and ecdc_23: ecdc_19 and ecdc_24.

MLVA was performed by each of the participants largely using their own protocols adapted from previously published assays [12,18,19]. All countries used a single multiplex PCR except three countries that used two separate multiplex PCR, two of which used the PulseNet protocol [18,19] and one an in-house protocol targeting five loci. Annealing temperatures ranged from 55 °C to 60 °C and were individually optimised for each laboratory. Primer concentrations were also individually optimised as per the recommendation of Nadon et al. [14].

Twelve of 14 laboratories used Applied Biosystems Genetic Analyzer (ABI) platforms (Foster City, California, US) for CE, one laboratory used the Beckman Coulter platform (Brea, California, US) and the remaining laboratory used both systems.

### Allele assignment

For the validation set, at the five respective loci in the order SENTR7; SENTR5; SENTR6; SENTR4; SE-3, each laboratory was requested to report the number of tandem repeats found and the fragment sizes used to determine them. Where no predominant peak was present at a locus, this was considered to be a null allele and reported as NA (no amplification at this locus).

Participants were free to use any local method for calculation of the number of repeat units from their obtained fragment sizes. A number of laboratories used a compensation matrix in Excel format originally developed for MLVA of *S.* Typhimurium by Larsson et al. [9] while others adopted the use of binned datasets with an expected range of fragment sizes suggested for each set of tandem repeats. The latter approach relied upon look-up tables with the allele size range being well-characterised for each of the five loci.

### Comparability analysis

The inter-laboratory comparability of the *S.* Enteritidis MLVA method was considered as adequate if more than 80% of the participating laboratories reported less than 5% discrepant MLVA type assignment for the blinded set of validation strains [14].

## Results

Of the 14 participating laboratories, eight reported expected profiles for all 22 validation strains and their 110 loci (Tables 2 and 3). Five reported expected profiles for 21 out of 22 validation strains and 109 of their 110 loci, although one of these laboratories reported all loci as expected when using another sequencing platform. One laboratory reported expected profiles for 20 out of 22 validation strains and 108 of their 110 loci.

**Table 3:** Capillary electrophoresis platforms, size markers, dye sets and proportion of loci reported as expected in the *Salmonella* Enteritidis MLVA inter-laboratory validation study, Europe^a^, 2015 (n = 14 participating laboratories)

Laboratory	Size marker	Dye set	Capillary electrophoresis	MLVA score^b^ (%)
A	GeneScan 600 LIZ	ABI G5	ABI 3130	100.0
B	GeneScan 600 LIZ	ABI G5	ABI 3130xl	100.0
C	GeneFlo 625 ROX	ABI D	ABI 3730xl	100.0
D	GeneFlo 625 ROX	ABI D	ABI 3130xl	99.1
E	GeneScan 1200 LIZ	ABI G5	ABI 3130xl	100.0
F	GeneScan 600 LIZ	ABI G5	ABI 3730xl	100.0
G	GeneFlo 625 ROX	ABI D	ABI 3730	99.1
H	GeneScan 600 LIZ	ABI G5	ABI 3500	98.2
I	CEQ DNA Size Standard Kit‑600	D2, D3, D4	Beckman Coulter GeXP	99.1
J	GeneFlo 625 ROX	ABI D	ABI 3130XL	100.0
K	Roche LIZ1200	Unknown	ABI 3730	99.1
L	GeneScan 600 LIZ	ABI G5	ABI 3130	100.0
M	GeneScan 600 LIZ	ABI G5	ABI 3500xL	100.0
N	CEQ 600-bp DNA size standard	D2, D3, D4	Beckman Coulter GeXP	100.0
N	GeneFlo 625 ROX	ABI D	ABI 3500	99.1

MLVA: multilocus variable-number tandem repeat analysis.

^a^ Fourteen laboratories from 11 European Union and European Economic Area countries and two laboratories from North America (Canada and the United States).

^b ^Percentage of loci correctly assigned out of a total of 110.

Two validation strains were excluded from the result analysis. Eight laboratories reported double peaks or finding two distinct MLVA profiles for ECDC3, and four laboratories reported more than one allele at the second locus, SENTR5, for ECDC21. As so many participants reported issues with these two strains, it is probable that they contained a mixed population. Those laboratories with greater experience of the MLVA process were still able to ascertain the correct profiles for these strains following purification and analysis of multiple colonies.

Sporadic deviations from the expected results in single loci were reported by six participants. For ECDC8, laboratory D reported one TR at the last locus, SE-3, while all other participants recorded the expected result of no amplification at this locus. For this same strain laboratory H recorded an additional two TRs at locus SENTR5; i.e. six TRs instead of the expected four. This was due to a conversion error in their results tables as the fragment size they recorded equated to four TRs and not six. Laboratory H also recorded an additional TR at locus SENTR5 for ECDC10. Again this would appear to be a conversion error as the correct fragment size for seven TRs was recorded. Laboratory K reported one less TR at locus SENTR7 for ECDC14, corresponding to a fragment size of 130.6 bp. For a fragment of this size, the result should have been recorded as three TRs and not two TRs so this was also likely a conversion error. Furthermore, Laboratory N reported six TRs instead of the expected eight TRs for SENTR5 locus of ECDC14. Laboratory G was the only participant to report a mixed population for ECDC4. For the two MLVA profiles they recorded for this strain, one profile equated to ECDC7 while the other profile was similar to that of ECDC4 apart from the loss of a TR repeat at locus SENTR4 i.e. three TRs instead of the expected four. Additionally, although Laboratory M reported what appeared to be a mixed population for ECDC11, they were still able to report the correct final MLVA profile.

Laboratory I initially reported difficulty using the calibration strains which resulted in a large number of erroneous results for all 24 validation strains. This was these participants’ first experience at setting up a MLVA protocol for *S.* Enteritidis and they were one of the few laboratories using a Beckman Coulter platform. Following feedback about these problematic results, Laboratory I carried out further optimisation of their PCR and CE protocols before resubmitting their results. This new set of results corresponded much more accurately to the expected results for the validation strains. Apart from the previously mentioned problems for ECDC3 and ECDC21, Laboratory I were unable to correctly amplify a fragment for SENTR4 of ECDC23. However, they did report this fragment correctly for ECDC17 which was the duplicate isolate of ECDC23.

Laboratory F initially reported consistently higher repeat numbers for SENTR7. However, these issues were resolved after adjusting the ranges for repeat number assignment using the calibration strain set.

### Comparison to PulseNet protocol for *S.* Enteritidis MLVA

Importantly, during this validation study it was noted that there were differences between the five-loci MLVA protocol [12] and the PulseNet protocols [18,19] in two alleles: compared with PulseNet results, the five-loci protocol gave consistently one less repeat number for SENTR4, and two repeat numbers less for SE-3. However, the issue was purely related to the result analysis since the raw data (measured fragment lengths) gave consistent results if the result analysis i.e. assignment of TR numbers was changed (raw data from the reference and validation strains obtained with PulseNet protocol analysed using conversion tables for the five-loci protocol).

## Discussion

The *S.* Enteritidis MLVA protocol targeting five loci was shown to produce internationally comparable results during the inter-laboratory validation study. More than 90% of the participating laboratories reported less than 5% discrepant MLVA profiles for the blinded set of validation strains. All 14 participating laboratories performed well, even those where experience was initially lacking in MLVA and fragment analysis technology. The most critical phase was the conversion of raw fragment length data to repeat numbers, an issue that the present inter-laboratory validation helped to standardise.

Following the proof-of-concept study published for *S.* Typhimurium MLVA [7], this study has likewise shown the efficacy of using calibration strains for MLVA of *S.* Enteritidis to minimise any differences in laboratory set-ups. While the general idea for multi-laboratory validation is not new [12,14,20], to our knowledge this is the first international, inter-laboratory study to verify the concept for this particular serovar.

Despite the wide variation in laboratory protocols, CE chemistries and level of experience in MLVA methods, all 14 participants demonstrated that they could correctly identify MLVA profiles with a minimum of 98% correct allele assignments for the validation strain set. Thirteen of the participants returned correct assignments for practically all of the 110 targeted alleles. 

Even with the lack of a standardised data analysis system, all laboratories were able to obtain comparable results for virtually all of the loci tested within the validation set. Six laboratories reported sporadic deviations from the expected results in single loci. In one of these laboratories, the MLVA method for *S.* Enteritidis had only recently been set up. Had they gained more experience in this method and made more rigorous TR assignments, this laboratory would have also likely identified all 110 alleles correctly. Likewise, for the other five laboratories with sporadic deviations, the importance of critically assessing data for each individual locus in comparison to the results corresponding to the other TR numbers in the same locus is highlighted, e.g. where SENTR5 is known to comprise of a 6 bp TR and a fragment size of 237 bp represents five TRs, then a fragment size of 231 bp should logically represent one less repeat i.e. four TRs. The conversion errors might be due to human error when converting raw data into TR numbers, but likely the absence of consolidated procedures for this critical step also plays an important role. To avoid the possibility of human errors, automated processing of the raw data to repeat numbers via dedicated software can be helpful. In addition, regular External Quality Assessments (EQAs) for MLVA for *S.* Enteritidis should be set up at the EU/EEA level to ensure that data remain comparable and consistent.

Where other differences were noted, they only occurred as single locus variants rather than gross deviations from the expected MLVA profile. The initial discrepancy in MLVA allele assignment in Laboratory I was caused mainly by difficulties in optimising the PCR and the lack of any significant prior experience in fragment analysis with the Beckman Coulter platform.

Although it has been previously recorded that some VNTRs are not entirely stable [21,22], Bertrand et al. have shown that there were no variations over time for the five MLVA loci chosen for *S.* Enteritidis following numerous serial passages of the organism [23]. From this present study, although it would appear that the stability of the number of tandem repeats in the MLVA loci is not in question, it is also not entirely unexpected to occasionally find a single locus variant among a large set of alleles. Within the blinded panel of validation strains, three isolates were represented twice to test for reproducibility and repeatability of the method. All laboratories correctly identified the replicates apart from one laboratory that could not verify a fragment for SENTR4 of ECDC23. This may have been due to the previously mentioned challenges this laboratory experienced trying to establish the methodology in the absence of deep-rooted knowledge or workflows for their MLVA system.

Based on previous studies, the discriminatory power of MLVA for *S.* Enteritidis has limitations. Bertrand et al. [23] concluded that one single MLVA profile represented more than a quarter of 1,498 *S.* Enteritidis strains isolated during 2007–2012 in Belgium. The most common MLVA types can be further divided in subgroups using phage typing and PFGE [12,23,24]. This indicates that MLVA should not be relied upon as a single typing method but complementary methods should be used in parallel for prevalent MLVA types. Furthermore, since MLVA schemes for *Salmonella* are serovar-specific, the method cannot fully replace PFGE. Subtyping methods based on next generation sequencing technologies show enormous potential. They have been shown to produce epidemiologically robust data also for *S.* Enteritidis with a superior discriminatory power compared with MLVA [24,25], but data standardisation and common nomenclature need to be agreed upon before the results can be used routinely for international comparisons [26,27]. Until then, MLVA could have a role in providing a common international strain nomenclature and providing an adequate typing method for laboratories that do not foresee moving to whole genome sequencing technology in the near future.

Even with the above-mentioned limitations, MLVA has already been shown to be a good candidate for performing *S.* Enteritidis surveillance at EU/EEA level [3], and it can only be beneficial to further this development to additional pathogens and on a global scale. Both PulseNet International and ECDC have already published suggested operating procedures for *S.* Typhimurium MLVA [9,28]. In addition, PulseNet International have also published MLVA protocols for *S.* Enteritidis [18,19] and verotoxigenic *Escherichia coli* O157 [29]. As discovered during our study, the five-loci MLVA protocol [12] and the PulseNet protocols for *S.* Enteritidis assign repeat numbers differently for loci SENTR4 and SE-3 although the raw data from the two protocols are consistent. This is due to the fact that PulseNet currently assigns alleles based on the calculated copy number, not the actual sequenced copy number. This should be remembered when exchanging data during international outbreak investigations to ensure a rapid, cooperative response, which is important for source tracing, particularly with the global food markets of today where cross-border action may be required [30].

Subtyping of *S.* Enteritidis is important for outbreak detection and timely provision of information for surveillance programmes such as TESSy and PulseNet International. The use of the nomenclature in this study is currently widely accepted within the EU/EEA as unambiguous when applied to MLVA of *S.* Enteritidis. As demonstrated by our study, even when multiple, only partially overlapping protocols are used in many different countries around the world, it is still possible to exchange data without rigid standardised methodology and equipment. To facilitate the set-up in laboratories with no experience in the method, the European Centre for Disease Prevention and Control (ECDC) has published a standardised protocol for *S.* Enteritidis MLVA [31]. *S.* Enteritidis MLVA data collection for EU/EEA countries has been started in TESSy in June 2016, enabling EU/EEA-wide analysis of *S.* Enteritidis MLVA data and multi-country cluster detection.
